# When Markets Shape AI Mental Health Self-Management Tools: Consequences for Serious Mental Illness

**DOI:** 10.2196/99143

**Published:** 2026-08-25

**Authors:** Lucy Smith, Yousef Yousef, Piotr Wasilewski, Hajira Dambha-Miller

**Affiliations:** 1 University of Southampton, Aldermoor SurgeryAldermoor Close, Southampton, England, S016 5ST, United Kingdom, 44 (0)23 8059 1759

**Keywords:** mental health, artificial intelligence, AI, digital tools, serious mental illness, digital mental health, clinical decision support systems, health equity

## Abstract

AI-enabled self-management health tools are increasingly promoted within health care policy as part of digital self-management models for mental health care. However, development is concentrated on scalable, low-intensity interventions for common conditions, such as anxiety and depression, rather than on populations with the greatest clinical need, such as those with serious mental illness (SMI). This includes schizophrenia-spectrum and bipolar disorders, which remain comparatively underserved, despite experiencing a disproportionate burden of morbidity and service use. However, the clinical features of SMI—comprising fluctuating symptoms, multimorbidity, and elevated risk—may limit the suitability of low-intensity AI-driven self-management tools designed for mild-to-moderate conditions. In this Viewpoint, we argue that the scarcity of AI-enabled self-management tools for SMI reflects a structural feature of current innovation systems rather than one of technical infeasibility alone. Market incentives, regulatory pathways, and fragmented research pipelines favor low-risk, high-volume populations, thereby limiting development for clinically complex groups. Addressing this unevenness is essential and will require upstream intervention, including targeted public funding, improved data infrastructure, and administrative frameworks that support safe innovation in high-risk populations. Embedding equity for SMI as a primary design requirement will be necessary to ensure that AI-driven mental health tools do not reinforce current inequalities.

## The Problem: AI Mental Health Tools Are Not Built for Serious Mental Illness

AI self-management tools are now a familiar part of digital mental health. They are used to track symptoms, deliver prompts, personalize advice, and simulate supportive conversations outside formal appointments. Within policy and industry, they are often presented as an effective response to rising demand and reduced workforce capacity [1]. However, while there is a growing body of research exploring AI-supported approaches for serious mental illness (SMI)—including supervised digital phenotyping, passive sensing, and relapse prediction systems—these remain largely at the feasibility or pilot stage, with limited evidence for large-scale implementation or integration into routine care.

Furthermore, the benefits of this wave of innovation are not being distributed evenly. In mental health, AI self-management tools are concentrated in lower-acuity conditions, especially anxiety and depression, where risk is lower and delivery models are more easily scaled. By contrast, people living with SMI, including schizophrenia-spectrum and bipolar disorders, remain comparatively underserved despite disproportionately high levels of morbidity, physical multimorbidity, and service use [2-4]. We argue that this is not an incidental omission. It reflects how AI mental health innovation is currently structured. Markets reward tools that can be deployed at scale, have a low regulatory burden, and are trained on accessible datasets. That logic favors mild-to-moderate conditions and works against SMI. The problem is not simply that too few tools are being designed for SMI, but that prevailing models of AI mental health self-management are poorly aligned with the clinical realities of SMI care [5,6]. This mismatch is reflected in both outcomes and evidence. Individuals with SMI die 10 to 20 years earlier than the general population, largely due to preventable physical conditions, and experience particularly high levels of physical multimorbidity [3]. Despite increasing investment, demand for mental health services continues to rise [7]. In contrast, the evidence base for AI mental health tools shows limited engagement with SMI populations. A meta-analysis of 35 studies found that AI-based conversational agents have been evaluated primarily for depression and anxiety, with minimal investigation of their effectiveness for SMIs, such as schizophrenia or bipolar disorder [8]. Studies are further characterized by short follow-up, limited external validity, and a lack of representation of underserved populations, such as those captured within the NHS England CORE20PLUS5 framework [8] (which identifies the most deprived 20% of the population, alongside additional inclusion health and clinically vulnerable groups), although equivalent disadvantaged populations are recognized across other health systems.

AI-enabled mental health tools are not a uniform category. They include chatbot-based interventions, symptom tracking apps, passive sensing systems, digital phenotyping platforms, and clinically integrated monitoring tools, each of which involves different assumptions about user autonomy, clinical oversight, safety, and regulatory classification.

## Why SMI Does Not Fit the Dominant Self-Management Model

SMI presents a different clinical problem from the conditions that dominate current AI self-management tools. Schizophrenia-spectrum and bipolar disorders are characterized by fluctuating symptoms, variable insight, and periods of impaired decision-making. Clinical risk is fluid and may escalate quickly. For example, during periods of emerging psychosis, individuals may experience reduced insight or increasing paranoia, which can affect how they interpret and interact with digital tools, including mistrust of prompts or disengagement from monitoring. Similarly, in bipolar disorder, shifts from depressive to manic states may result in periods of high engagement followed by abrupt disengagement or impulsive use, undermining assumptions of consistent, stable interaction over time.

Early signs of deterioration may be difficult to detect without continuous monitoring and clinical context. Care depends on ongoing assessment, coordination across services, and timely reaction to emerging risk [2,3]. Most AI self-management tools are designed for stable users with steady engagement. They assume that delayed or incomplete responses carry limited clinical consequences. These distinctions are important, as different types of AI systems place varying demands on user engagement and clinical supervision, with fully autonomous tools posing different risks from clinician-integrated monitoring approaches. These assumptions do not hold in SMI, where missed or misinterpreted signals can cause delayed intervention and increased risk. This misalignment is reflected in how tools are developed and evaluated. Existing AI self-management tools have been developed and evaluated almost entirely for physical chronic conditions rather than SMI, which limits the applicability of findings to SMI populations [5]. Tools are built and tested in settings that do not capture the variability, risk, and multimorbidity seen in routine care. Key functions required for SMI self-management are often absent. These include relapse detection, medication adherence support, and escalation pathways linked to clinical services. The dominant AI self-management model is built around a different clinical use case and does not align with the requirements of SMI care. Existing key functions could also cause harm for those with SMI, including false reassurance during deterioration or the potential for systems to unintentionally reinforce paranoia or maladaptive beliefs in vulnerable populations. AI tools based on large language models require patients with SMI to self-advocate, either through text or voice automation. For people with SMI who experience cognitive distortion, flattened mood, or delusions, this is particularly challenging [9]. Research shows that for people with paranoia, using digital interventions is associated with fear of being watched or monitored—AI would likely exacerbate this anxiety by its very nature [10].

There are several emerging research programs attempting to address this issue through passive sensing, relapse prediction, and clinician-supervised monitoring systems in psychosis and bipolar disorder. For example, mindLAMP, an open-source smartphone app used passive sensing (geolocation, accelerometer, and screen state) and active data (surveys) to demonstrate relapse prediction modeling for people with schizophrenia, with the authors highlighting the need for future work to *“*elucidate how anomaly detection models can be integrated effectively into clinical care” [11].

## Innovation Follows Markets, Not Burden of Need

AI self-management tools are shaped by commercial and regulatory incentives that prioritize scalability, low risk, and predictable returns. Conditions including mild depression and anxiety align with these requirements. They are common, relatively stable, and associated with populations that tend to engage with digital tools [12]. The rapid growth of this market further incentivizes development for these groups [13]. This enables rapid iteration and reduces uncertainty for developers and investors. Products can be deployed at scale with limited clinical oversight and lower regulatory burden. SMI populations do not align with these conditions. They are more heterogeneous, experience higher levels of clinical risk, and commonly face barriers to digital access. Tools designed for SMI require closer integration with clinical services, including monitoring, escalation, and continuity of care. This increases development difficulty and regulatory exposure. Current tools reflect this pattern. Apps including Wysa [14] and Woebot [15] deliver chatbot-based support for mild-to-moderate symptoms and demonstrate high levels of engagement [16]. These tools are often positioned as wellness apps and operate outside stricter medical device regulations. This allows faster development and wider deployment. For SMI, this approach is insufficient. Supporting relapse prevention, medication adherence, and risk monitoring requires clinical validation and system integration. A scoping review of AI self-management tools for chronic conditions found that emotional self-management was the least addressed of the 3 core self-management tasks, and that the tools identified targeted physical conditions rather than SMIs, such as psychosis or bipolar disorder [5]. Innovation appears to concentrate on populations that are easier to serve. This results in a concentration of tools in lower-acuity conditions and limited development for SMI.

## The Research Pipeline Reinforces Exclusion

The development of AI mental health tools depends on the availability of suitable data. For SMI, these data are limited. Individuals with SMI are frequently excluded from clinical research, which reduces the availability of representative datasets for model development. Exclusion occurs through both study design and eligibility criteria. Psychiatric conditions are excluded from approximately half of clinical trials [17]. Exclusion rates for substance use disorders range from 64% to 96% [18]. Co-occurring SMI is often excluded directly or through broader psychiatric exclusion criteria. Moral concerns around consent are frequently cited, although present frameworks support inclusive approaches [19]. These practices affect the quality and structure of available data.

SMI datasets are smaller, more heterogeneous, and affected by missingness and attrition [20]. There are also important differences in symptom expression that must be considered in the design of data-driven AI systems, given that these models rely on predictive algorithms trained on observed patterns. SMI-related data are inherently difficult to model due to symptom instability, diagnostic overlap, fluctuating adherence, comorbid substance use, and inconsistent outcome definitions. In practice, schizophrenia spectrum disorders, bipolar disorder, schizoaffective disorder, and co-occurring substance use disorders present major differences in symptom trajectories, treatment patterns, digital engagement, and data reliability. For example, schizophrenia spectrum disorders tend to have consistent symptoms from baseline reporting that require continual maintenance treatment [21]. For bipolar disorder, symptoms are episodic, oscillating between severe mania and depressive symptoms, with periods of relatively stable euthymic functioning in between [21]. For co-occurring SMI with substance abuse disorders, drug use can destabilize all other disorders leading to relapse and worse functioning [21]. Taken together, these differences highlight the limitations of treating SMI as a single analytical category and underscore the need for more diagnosis-sensitive approaches to data collection, model development, and evaluation.

**Figure 1.**
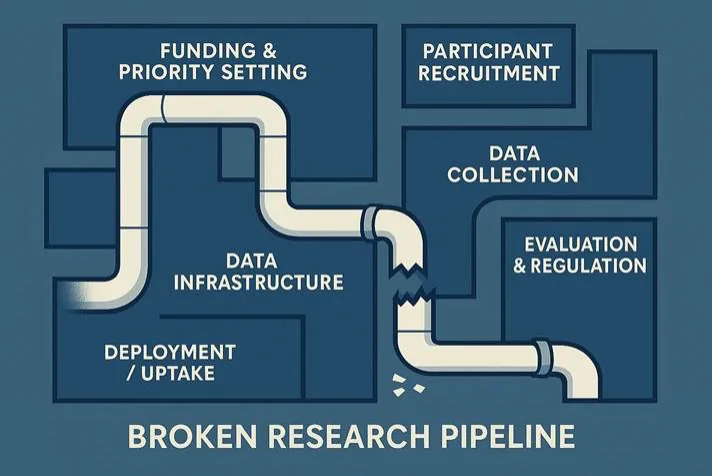
Visualization of the broken research pipeline that drives the exclusion of people with serious mental illness from the research needed to develop clinically safe, AI-driven self-management mental health tools.

Furthermore, data are fragmented across primary, secondary, and community care settings and commonly rely on unformatted clinical records [22]. Underserved populations, including individuals experiencing homelessness, are underrepresented despite a higher prevalence of SMI [6,23,24]. These limitations carry direct implications for AI development. Models developed on incomplete or unrepresentative data are more likely to produce unreliable outputs. In SMI, this increases the chance of missed deterioration, incorrect classification, and inappropriate recommendations [25]. The research pipeline likely influences what can be built. Limited and disjointed data reduce confidence in model effectiveness and increase perceived development risk. This also discourages investment in SMI tools and reinforces the concentration of innovation in lower-risk populations. We argue that this may create a feedback loop in which market incentives influence which research is funded, which appears to shape the research pipeline and may further reinforce existing market priorities. Figure 1 presents a conceptual model illustrating these potentially reinforcing relationships.

The pipeline illustrates how exclusion occurs across a series of interconnected stages: (1) funding and priority setting, where investment is directed toward low-risk, scalable conditions, limiting resources allocated to complex SMI populations; (2) data infrastructure and deployment and uptake, where funding decisions shape the development of data systems, platforms, and implementation models that are often not designed for or accessible to people with SMI; (3) participant recruitment, where these structural and design constraints reduce inclusion of individuals with SMI, particularly those from underserved groups; (4) data collection, resulting in smaller, fragmented, and unrepresentative datasets that fail to capture the heterogeneity and instability of SMI; and (5) evaluation and regulation, where current frameworks do not consistently require inclusion of the full spectrum of SMI diagnoses or underserved populations, allowing tools to be validated on narrower, lower-risk groups. Together, these stages may form a reinforcing cycle in which early funding decisions shape downstream data infrastructures and deployment models, which could in turn influence who is recruited and what data are collected, ultimately limiting the evidence base used for evaluation and regulation and perpetuating the exclusion of SMI populations from AI-enabled mental health innovation.

## Equity Cannot Be Retrofitted

SMI is more prevalent among underserved populations and is determined by socioeconomic, cultural, and structural factors [24]. These include higher exposure to deprivation, unstable housing, and barriers to accessing care, all of which affect both illness burden and engagement with services [4,24]. In addition, help seeking in SMI is frequently mediated through family, community, or religious networks and influenced by stigma and barriers to services [6,26]. Effective self-management tools must reflect social context, patterns of help seeking, and access to care [27].

Engagement with digital tools is heterogeneous. Universal assumptions about individual, self-directed use do not reflect how access to digital support is socioculturally contextualized across settings. However, development processes do not consistently incorporate these factors; evidence from digital mental health research shows that underserved groups are consistently underrepresented in both trials and implementation studies [8]. Addressing this in practice would involve embedding meaningful co-design processes with people living with SMI from underserved groups across the entire product development cycle. Consideration should also be given to the inclusion of the wider social networks who support the user, including informal caregivers and wider community support organizations, such as religious groups, rather than assuming individual, independent engagement, to capture the wider contextual mechanisms that shape support trajectories for people with SMI. It must also be noted that some individuals living with SMI may actively prefer human-supported or relational models of care over self-management paradigms, independent of technological accessibility or design limitations. Coproduction with nonadopters and reluctant adopters would help to identify barriers to use, which could lead to the development of acceptable hybrid alternative forms of self-management support that could be used alongside or instead of AI tools. These priorities are consistent with emerging international consensus guidance, which emphasizes coproduction, multidisciplinary collaboration, and the integration of digital tools within existing care systems, alongside careful consideration of ethical risks and real-world implementation challenges [28].

However, this may be hampered by the challenges of meaningful co-design with underserved populations, which requires time, resources, and specialist expertise. Consequently, co-design activities are often deprioritized in commercial development, where products are designed for populations that are easier to recruit, retain, and scale. This risk reinforces existing inequities [29], especially if tokenistic approaches are taken which do not capture wider contextual mechanisms in a way that is clinically relevant.

The structural timing of design decisions shapes who benefits from these tools. Features, data inputs, and user pathways are established at the outset. Once systems are built and deployed, adapting them to excluded populations calls for extensive redesign and additional validation [20]. In practice, this means that populations with the highest burden of SMI experience the longest delays in reaping the benefits of innovation. Equity needs to be addressed at the point of design. Delayed consideration limits effectiveness and slows access for populations with the greatest need.

## Rethinking the Development Pathway

Addressing the imbalance requires intervention earlier in the development pathway. Decisions about target populations, acceptable risk, and product design are made at early stages and shape what can be developed and scaled. Public funding can shift the risk profile of innovation. Operationally, upstream intervention could include ring-fenced funding calls specifically requiring inclusion of SMI populations, staged funding tied to progression from feasibility through real-world implementation, and requirements for clinical service integration from early phases of development. For example, funding criteria could prioritize tools that demonstrate linkage to routine care pathways, such as automated relapse alerts routed to community mental health teams or integration with electronic health records.

While the following examples draw on United Kingdom–based programs, they are intended as illustrative case studies of a broader class of policy mechanisms that could support innovation in high-risk and underserved populations across different health systems and can be applied internationally. Practical examples include programs such as the National Institute for Health and Care Research (NIHR) Invention for Innovation (i4i) and the AI in Health and Care Award, which support technologies from early feasibility to real-world evaluation using staged progression linked to clinical and technical milestones [30]. These models allow evidence generation in areas where commercial return is uncertain. Mission-oriented approaches, including those used by the Advanced Research and Invention Agency (ARIA), support high-risk and high-uncertainty innovation and provide a mechanism to prioritize areas of unmet clinical need [31].

Data infrastructure is also critical. In SMI, relevant data are fragmented across primary, secondary, and community care and are not consistently captured in structured formats [3,20,22]. Interoperable systems based on standards, including Fast Health Interoperability Resources (FHIR), can support linkage across these settings and improve access to more representative datasets [22]. This can reduce barriers to development, particularly for smaller or equity-focused teams.

Infrastructure alone is not sufficient. Data quality, governance, and controlled access are required to support safe and reliable model development. Improved data infrastructure would involve practical steps such as creating linked datasets across primary, secondary, and community care; developing secure data environments that allow controlled access for model development; and standardizing data capture using interoperable formats, such as FHIR. In addition, establishing longitudinal cohorts of people with SMI with consent for data linkage and AI development could directly address current gaps in representativeness and data continuity. Without these safeguards, improvements in data availability are unlikely to translate into clinically usable tools. Appraisal frameworks also need to reflect the realities of SMI care. Current approaches often focus on engagement or short-term symptom change. For SMI, evaluation needs to include safety, relapse detection, escalation, and integration with clinical services. Tools must demonstrate their ability to support care in conditions characterized by instability and multimorbidity. Intervention at this stage has the greatest impact. Once products reach later stages of development, market incentives and regulatory pathways narrow the range of viable applications. Early investment and design choices determine which populations are included and which are left behind.

## Limitations

We acknowledge that this Viewpoint is based predominantly on United Kingdom and North American evidence, which may not reflect the impact of commercialization on the development of AI tools for SMI within collectivist cultures. Our work does not draw on a systematic review methodology; thus, the recommendations made represent normative propositions rather than empirically validated conclusions.

## Conclusions

AI-supported care spans a spectrum from fully autonomous self-management tools to clinician-integrated augmentation models, with distinct implications for safety, feasibility, and clinical integration in SMI populations, and these models are increasingly visible within health systems and policy. However, there is a mismatch between technological development and the realities of clinical care. Importantly, different models of AI-supported care carry distinct implications for SMI. Fully autonomous self-management tools, which assume independent and stable user engagement, may be poorly suited to populations with fluctuating insight and clinical risk. In contrast, clinician-integrated or augmentation models—such as hybrid monitoring systems linked to care teams—may offer more feasible and safer pathways, though they require greater system integration and resources.

This Viewpoint proposes that market incentives, research priorities, and data infrastructure may interact in ways that contribute to a broken research pipeline, with the potential to exacerbate rather than reduce health inequities in SMI populations. While developers need incentives to fund research that broadens population reach, health care policy needs to drive this through the creation of funding calls that emphasize the need for equity across the development decision-making cycle, promote the safety of tools that reduce clinical risk across differing SMIs, and incorporate hybrid approaches for reluctant or late digital adopters. To address the feedback cycle driving the broken research pipeline, calls should promote meaningful collaboration between academia and industry. Emerging systems, including digital phenotyping and clinician-integrated monitoring platforms, such as mindLAMP, demonstrate that alternative approaches to AI-supported care for SMI are feasible and offer promising directions for future innovation. AI mental health tools have the potential to support earlier intervention and improve continuity of care—realizing this potential requires better alignment of innovation with clinical needs.

## Abbreviations

ARIA: Advanced Research and Invention Agency
FHIR: Fast Health Interoperability Resources
i4i: Invention for Innovation
NIHR: National Institute for Health and Care Research
SMI: serious mental illness
